# User-Independent Hand Gesture Recognition Classification Models Using Sensor Fusion

**DOI:** 10.3390/s22041321

**Published:** 2022-02-09

**Authors:** Jose Guillermo Colli Alfaro, Ana Luisa Trejos

**Affiliations:** 1 School of Biomedical Engineering, Western University, London, ON N6A 5B9, Canada; jcollial@uwo.ca; 2 Department of Electrical and Computer Engineering, Western University, London, ON N6A 5B9, Canada

**Keywords:** body–machine interfaces, wearable devices, electromyography, sensor fusion, user-independent classification

## Abstract

Recently, it has been proven that targeting motor impairments as early as possible while using wearable mechatronic devices for assisted therapy can improve rehabilitation outcomes. However, despite the advanced progress on control methods for wearable mechatronic devices, the need for a more natural interface that allows for better control remains. To address this issue, electromyography (EMG)-based gesture recognition systems have been studied as a potential solution for human–machine interface applications. Recent studies have focused on developing user-independent gesture recognition interfaces to reduce calibration times for new users. Unfortunately, given the stochastic nature of EMG signals, the performance of these interfaces is negatively impacted. To address this issue, this work presents a user-independent gesture classification method based on a sensor fusion technique that combines EMG data and inertial measurement unit (IMU) data. The Myo Armband was used to measure muscle activity and motion data from healthy subjects. Participants were asked to perform seven types of gestures in four different arm positions while using the Myo on their dominant limb. Data obtained from 22 participants were used to classify the gestures using three different classification methods. Overall, average classification accuracies in the range of 67.5–84.6% were obtained, with the Adaptive Least-Squares Support Vector Machine model obtaining accuracies as high as 92.9%. These results suggest that by using the proposed sensor fusion approach, it is possible to achieve a more natural interface that allows better control of wearable mechatronic devices during robot assisted therapies.

## 1. Introduction

Recently, robot rehabilitation therapy has shown its greatest potential as a complementary method to traditional rehabilitation techniques. In order for robot-assisted therapies to be effective, patients must feel the interaction with the robotic device in a way that feels natural to them, while at the same time, receiving assistance from the robot based on their performance during the rehabilitation session [1]. To address this issue, gesture recognition has been considered as a possible solution for human–machine interface applications [2,3], being electromyography (EMG) the type of signal most commonly used in such applications [4]. By using EMG data, a classification model can be constructed to identify the intended gesture, and then control the behavior of the robotic device. To effectively use gesture recognition-based interfaces during rehabilitation sessions, it is necessary to develop strategies aimed towards a user-independent recognition system. In doing so, the deployment of robot-assisted therapies would be facilitated by using a system that can be adapted to new patients [5,6]. Moreover, this technique could be extended to reduce calibration times of each user by adapting an existing classification model based on the improvement obtained during the rehabilitation sessions.

To achieve this goal, several studies have tried to implement user-independent classification algorithms for gesture recognition. For example, Huang et al. [7] proposed the use of a smaller set of data obtained by extracting clusters of data points with similar characteristics in a pretrained classification model. Then, this model updated itself every time a new sample was ready to be classified. Likewise, Tommasi et al. [8] defined an adaptive classification model that considered a linear combination of existing pretrained models to classify grasping motions. A similar approach was adopted by Matsubara et al. [9] by proposing an EMG model formed by two types of factors: subject-dependent factors and motion-dependent factors. By separating these factors, they were able to create a user-independent classification model for new subjects.

Even though EMG-based user-independent classification models have great potential due to EMG signals being rich in information about muscle activity, EMG has a low signal to noise ratio (SNR), and because some patients may have limited motor abilities, these signals may be difficult to use during robot-assisted therapies. However, when used in combination with other types of sensors, it is possible to compensate for these disadvantages. This is known as sensor fusion, as it combines information from different sources to obtain a better understanding of the actions being performed [10]. One type of sensor that can be used in sensor fusion are inertial measurement units (IMU), which are formed by the combination of an accelerometer and a gyroscope. An advantage of these sensors is that they can provide information about the kinematics of human motion depending on their location on the body, and when used in combination with EMG data, they can improve the classification of gesture-based control algorithms [3,11]. Other advantages of using EMG–IMU sensor fusion include the ability to quantify motor function of stroke patients [12], to improve the control of prosthetic devices [13,14], and to account for postural changes when performing hand gestures [15,16]. Although promising, the use of EMG and IMU-based sensor fusion has not been fully explored in the area of user-independent classification algorithms. Therefore, in this paper, a sensor fusion technique that combines EMG and kinematic data coming from an IMU is proposed. It is expected that by adding another source of data, the efficacy of existing user-independent classification models can be enhanced. The remainder of this paper is divided as follows: Section 2 presents the methods of this study, including participant recruitment, the experimental protocol, and data processing. In Section 3, the user-independent classification models evaluated are presented. Finally, Section 4, Section 5 and Section 6 show the results, discussion, and conclusions, respectively, and some recommendations for future work.

Preliminary results of a previous version of one user-independent classification method described in this paper were published as a conference paper in [15]; however, this paper improves on the methods presented before including an increased number of participants recruited, as well as the addition of a statistical analysis. Furthermore, this paper focuses on the enhancement of multiple user-independent classification models —including the model presented in the preliminary study— using sensor fusion, and also discusses the performance of the models when compared against each other. The implementation of the sensor fusion technique in different user-independent classification models is presented in the following section.

## 2. Materials and Methods

### 2.1. Participant Recruitment

To create a user-independent classification model based on EMG and IMU sensor fusion, an experimental study was conducted using real user data. EMG and IMU data collection began following approval from the Human Research Ethics Board at Western University (Project ID: 112121). Data were collected from 24 healthy subjects over the age of 18, with no previous injuries of the shoulder, elbow or wrist, and no neurological disorders. Upon enrollment, written informed consent was given by each participant before continuing to the experimental protocol phase. Table 1 shows a summary of the demographics of the participants.

### 2.2. Experimental Protocol

**Figure 1:**
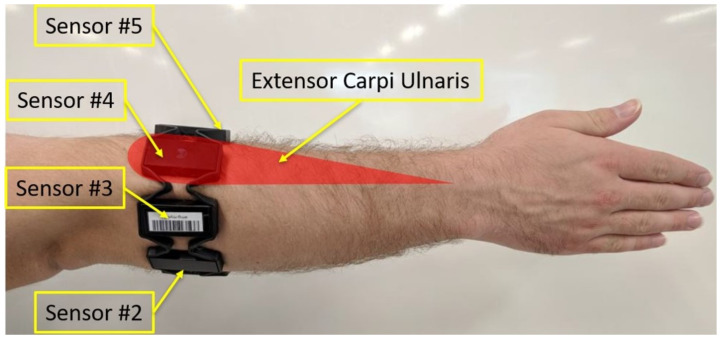
Placement of the Myo Armband on the user’s dominant arm. The IMU is located within Sensor 4.

Following consent, participants were asked to wear the Myo Armband on their dominant arm. The Myo is a gesture recognition armband which comprises eight dry EMG sensors and one 9 degree-of-freedom (DOF) IMU [17]. The armband was placed approximately one inch distally from the elbow joint of the participant’s dominant arm, following the instructions of the armband manufacturer. In order to avoid further variability between subjects, the fourth sensor of the Myo Armband was positioned above the Extensor Carpi Ulnaris muscle (Figure 1). This was done so that the IMU sensor, which was located within Sensor 4, was located on the dorsal side of the forearm as recommended by Höglund et al. [18].

### 2.3. Gestures

**Figure 2:**
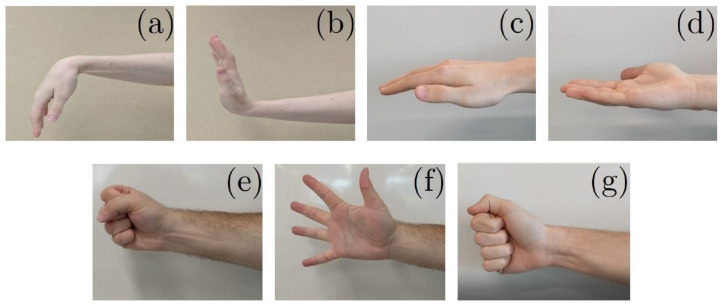
Wrist and finger motions that were recorded. (**a**) Wrist Flexion (WF). (**b**) Wrist Extension (WE). (**c**) Wrist Pronation (WP). (**d**) Wrist Supination (WS). (**e**) Hand Closed (HC). (**f**) Hand Open (HO). (**g**) Key Pinch (KP).

After putting on the Myo Armband, participants were instructed to perform seven hand gestures (Figure 2). These seven gestures were selected based on a preliminary study [15], in which an artificial neural network was used to classify 10 gestures commonly used during activities of daily living. The results of this preliminary study helped optimize the gesture set, as three out of the 10 gestures showed a similar motion pattern due to their motions being controlled by the same group of muscles.

**Figure 3:**
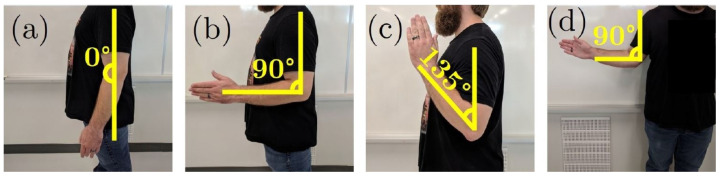
Arm positions used during data acquisition. (**a**) Forearm at full extension ($0^{\circ }$). (**b**) Forearm flexed at $90^{\circ }$. (**c**) Forearm flexed at $135^{\circ }$. (**d**) Forearm at $90^{\circ }$ flexion while externally rotating the shoulder through a comfortable range of motion.

Four trials were considered during the data collection phase. Each trial consisted in performing all seven gestures in a different arm position (Figure 3). This was done to allow the user-independent classification model to generalize better to unseen data, since it has been proven that changing the arm position affects the classification performance [19]. During each trial, each gesture was performed ten consecutive times at a moderate force level, and held for five seconds, with three seconds of resting time between repetitions. The order in which each gesture was performed was randomized in each trial for each participant. The rationale behind using 10 repetitions per gesture is explained with more details in Section 3. On the other hand, the purpose of holding the gesture for five seconds was to obtain as many data points as possible during data segmentation, in order to improve the performance of the user-independent classification methods. Finally, each trial was video-recorded to review the motions performed by the participants in case any abnormalities in the data were found during the analysis.

### 2.4. Data Acquisition and Processing

EMG data were recorded at a sampling frequency of 200 Hz, whereas IMU data were sampled at 50 Hz. Then, these data were streamed via Bluetooth 4.0 to a computer running a custom data acquisition GUI developed in MATLAB R2017b using the App Designer Toolbox and the Myo SDK MATLAB Mex Wrapper Toolbox [20].

For the EMG, the DC offset was removed, followed by the removal of the power line noise interference using a 60 Hz notch filter. Finally, the signal was filtered using a 4th order Butterworth high-pass filter with a cut-off frequency of 20 Hz. For the IMU, the accelerometer and gyroscope data were smoothed using a 4th order Butterworth band-pass filter with cut-off frequencies of 0.2 Hz and 15 Hz.

After processing the data, both EMG and IMU signals were divided into segments for future feature extraction. To facilitate this segmentation, data from the IMU were upsampled from 50 Hz to 200 Hz using a cubic spline, so that segments from both datasets had an equal number of samples. Then, sections of the EMG and IMU data collected when the gesture motion was being performed, were identified using the following procedure. First, in order to improve the accuracy detection of the motion onset, each EMG channel was conditioned with the Teager–Kaiser energy operator (TKEO), and then passed through a 4th order Butterworth low-pass filter with a 50 Hz cut-off frequency, before it was finally rectified. The TKEO is defined as follows [21]:

(1) $$\Psi \left(x_{i}\right)=x_{i}^{2}-\left(x_{i+1}\times x_{i-1}\right),$$

where $x_{i}$ represents the *i*th EMG sample value.

Then, the average absolute value of all of the channels for each sample was computed using the following equation:

(2) $$\Psi_{avg}\left(x_{i}\right)=\;\frac{1}{n}\;\sum_{j=1}^{n}\left|\Psi_{j}\left(x_{i}\right)\right|,$$

where *n* is the number of channels. Finally, $\Psi_{avg}$ was smoothed using Equation (3), with a window size W=60, to obtain its root mean square $\Psi_{rms}$,

(3) $$\Psi_{rms}\left(x_{i}\right)=\sqrt{\frac{1}{W}\sum_{j=i}^{i+W-1}\Psi_{avg}^{2}\left(x_{j}\right)}.$$

The motion onset and offset were obtained from $\Psi_{rms}$ using a variation of the double threshold technique proposed in [22]. The onset threshold was set to 20% of the average of all the peaks (local maximas), whose values were above 10% of the global maximum of $\Psi_{rms}$. Then, the offset threshold was set to 60% of the onset threshold. These percentages were determined experimentally. Afterwards, the indices of $\Psi_{rms}$ where the onset and offset occurred were taken and matched to every channel of the EMG signal. Using this technique, the active region of 72.51% of the datasets was successfully detected. The rest of the signals had their active region manually selected, as multiple onset and offset points were detected by the algorithm. Therefore, the active region of these signals was obtained by visually inspecting the signal, and then selecting the onset and offset index values. After obtaining the onset and offset indices of all of the EMG signals, they were matched to the upsampled IMU data. Finally, following the recommendations in [23], each active segment from the EMG and IMU data was divided into overlapping windows of 250 ms with 50% overlap, yielding 37 segments of data per gesture.

### 2.5. Feature Extraction

Following data segmentation, time domain features were extracted from each window of the EMG and IMU data. The following time domain features [24] were extracted from each EMG channel: the mean absolute value (MAV), mean absolute value slope (MAVS), waveform length (WL), 4th order auto-regressive coefficients (AR) and zero crossings (ZC). The following features were extracted from each axis of the IMU’s accelerometer and gyroscope: MAV and WL.

The result was a vector of 64 features ([4 features + 4 AR coefficients] × 8 channels) for each window of the EMG data, and a vector of 12 features (2 features × 3 accelerometer axes + 2 features × 3 gyroscope axes) for each window of the IMU data. From these feature vectors, two datasets were developed. The first one was formed by the 64 features extracted from each window of the EMG data, and the second dataset contained all 64 EMG features, plus the 12 extracted from the IMU data. In the case of the second dataset, a feature-level fusion approach was employed by concatenating each feature vector to form a single vector of 76 features. By using this fusion level, correlated features could be detected better during the feature reduction phase [25].

### 2.6. Cross-Validation Sets

Before implementing the classification methods, five cross-validation folds were created from 22 participants using a 5-fold cross-validation method. Although data from a total of 24 participants were collected, data from two participants (Subject 8 and Subject 21) had to be removed due to an improper execution of the gestures during the data collection phase. This was identified from the data and confirmed when watching the videos of the trials. Furthermore, each subject was randomly allocated to a cross-validation fold, and then training of the classification methods happened over five iterations using the data from four different folds (the training folds) during each iteration. On the other hand, data from each subject in the unused fold (the testing fold) during each iteration were used to test the classification methods. It is important to note that on average, each subject provided around 10,360 data samples (7 gestures × 37 segments of data × 10 repetitions × 4 arm positions).

## 3. User-Independent Classification Methods

After creating the cross-validation sets, three user-independent classification methods were used to classify the datasets obtained in Section 2.5. Two of these methods were briefly introduced in Section 1 and include the Adaptive Least Squares Support Vector Machines (LS-SVM) [8] and the Bilinear Model-based classification method [9]. The third classification method was implemented using a Multilayer Perceptron (MLP) Network, which is a type of artificial neural network (ANN). This method has been used in the past [26] to classify unseen data that are not easily linearly separable, e.g., EMG data. The decision to include a simple MLP Network in this study was based on the results from a previous pilot study [15].

It is important to mention that each method has its minimum requirements of repetitions per gesture to work correctly. In the case of the Adaptive LS-SVM and the Bilinear Models-based classifiers, the minimum number of repetitions per gesture is one. However, having 10 repetitions per gesture allows some of the user-independent classification models used in this study to perform better. For example, because the working principle of the adaptive LS-SVM is based on updating a predictive model that has a similar data distributions with new data, having more repetitions per gesture increases the chances of finding said predictive model. Similarly, for the Bilinear Models-based classifier, having more repetitions per gesture allows the extraction of better motion-dependent factors during the creation of the bilinear models. The implementation these classification methods is explained below.

### 3.1. The Adaptive LS-SVM

After extracting features from the EMG and IMU signals, the first user-independent classification method implemented was the Adaptive LS-SVM. This method was first proposed by Tommasi et al. [8] and consisted of adapting the information of past users to reduce the training time for new users. This adaptation process was performed by taking *N* number of pretrained predictive models (PM), each one corresponding to *N* different subjects, and then using one of these PMs as a starting point to train the data of a new subject N+1 (for more information about the mathematical principles behind the Adaptive LS-SVM, see [8]). Knowing that anatomical similarities exist when performing specific motions, it is fair to assume that among different PMs from multiple subjects, at least one could be used as a reference point to built a new PM for a new user. By adapting the PM parameters during the training phase of a new subject, it is possible to obtain a new PM using a portion of data instead of just using raw new data. The general process can be described as follows. First, for a group of subjects S1 to S*k*, a PM is created for each subject. Then, for a new Subject S*n*, data are prepared by normalizing and/or reducing them as necessary. Then, following standard machine learning procedures, a portion of the data of S*n* is separated so that it can be used later to test the performances of a new Predictive Model PM*n*. The remaining data of Subject S*n* are predicted using each PM of Subjects S1 to S*k*. The main idea of this step is to find the PM that predicts the data form Subject S*n* most accurately. In other words, the best PM will be the one that obtains higher accuracies when predicting data from S*n*, regardless on how good or bad is its performance. After finding the best performing model, its parameters are adapted to create a new Predictive Model PM*n* that can be used to predict data of Subject S*n* with better performance. This new Predictive Model PM*n* is tested using the portion of data separated mentioned before.

**Figure 4:**
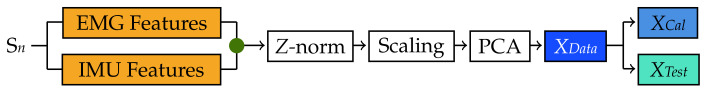
Training flow for the Adaptive LS-SVM classification method during each cross-validation iteration using both EMG and IMU data. A similar procedure was used for the EMG only dataset. Data from Subject S*n* in the cross-validation testing fold (orange) are split into *XCal* and *XTest*, which are then used to calibrate and test the classification models in Figure 5. The green dots represent a feature-level fusion.

**Figure 5:**
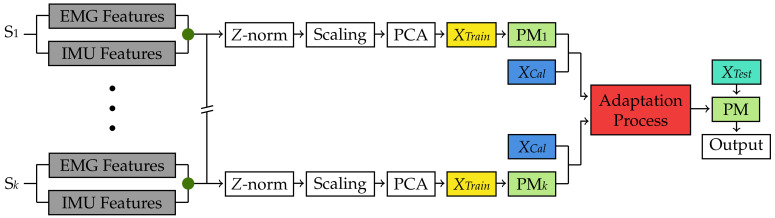
Testing flow for the Adaptive LS-SVM classification method during each cross-validation iteration using both EMG and IMU data. A similar procedure was used for the EMG only dataset. A predictive model (green) is created for each Subject S1 to Subject S*k*. Each predictive model is then used to classify *XCal* from Subject S*n* in the iteration training fold and then adapted using the procedure proposed in [8] (red). The adapted predictive model is tested using *XTest* (aqua) from Subject S*n*. The green dots represent a feature-level fusion.

Therefore, to implement this method, features from the EMG, and EMG and IMU datasets from each of the 22 subjects were standardized using the *Z* normalization, so that after normalization, data had a mean equal to zero and a standard deviation equal to one. Then, the normalized features were further scaled to the range of ±1, as this method is based on a margin classifier (i.e., LS-SVM). After feature normalization and feature scaling, these features were reduced using the principal component analysis (PCA) procedure with the singular value decomposition (SVD) algorithm to speed up the learning process (Figure 4 and Figure 5). A total of 17 principal components were used, so that at least 95% of the data variance was retained. After this data preparation, the Adaptive LS-SVM was implemented using the procedure described below.

First, during each cross-validation iteration, a LS-SVM PM was created for each subject S1 to S*k* in the training folds using their reduced data *XTrain* (Figure 5). Then, each subject in the testing fold had their reduced data *XData* divided into two smaller subsets *XCal* and *XTest* (Figure 4). For a subject S*n* in the testing fold, *XCal* was used to test each of the *k* PMs trained from subjects in the training folds to find the one that classified *XCal* data with the least error rate. However, given that Tomassi et al. found that each pretrained PM could classify different actions with better results than others, the classification outputs of the PM when classifying *XCal* were weighted by a parameter β found using the Projected Sub-gradient Descent algorithm proposed in [8]. This process is called the adaptation process (Figure 5), and it allows for the creation of a unique PM for Subject S*n* in the testing fold.

Because the idea of the adaptive LS-SVM is to create a new PM for a new user by using the minimum amount of collected data, *XCal* was formed by data corresponding to two random repetitions of each gesture. This was based on the assumption that because subjects were making the same gestures, the data distribution of new data samples was at least close to the data distribution of the already created PMs [8]. Therefore, the number of repetitions used to form *XCal* was a trade off between using the minimum number of data samples, and increasing the chances of finding a similar data distribution among the existing PMs. This number was found experimentally. Finally, the adapted PM for Subject S*n* in the testing fold was tested using *XTest*.

### 3.2. Bilinear Model-Based Classifier

After implementing the Adaptive LS-SVM, the next classification method explored was the one proposed by Matsubara et al. [9]. In their study, Matsubara et al. modeled the EMG signal collected from multiple channels *k* as a symmetric bilinear model, which was defined as follows:

(4) $$\Psi_{k}=z^{T}\cdot W_{k}\cdot x,$$

where $\Psi_{k}$ represents the EMG signal of channel *k*, $z\in R^{I\times 1}$ and $x\in R^{J\times 1}$ indicate the style (subject-dependent factors that make EMG signals different for each person) and content (motion-dependent factors) vectors of channel *k*; and $W\in R^{I\times J}$ is the weight parameter matrix of the bilinear model. The idea behind representing the EMG signal as a bilinear model is to find the style and content factors, and the weight parameter matrix to extract the motion-dependent factors (as they will be the same across multiple subjects), and then use them as inputs for a classification model (e.g., MLP Networks, SVM, and others). Similarly to the Adaptive LS-SVM, the main idea behind the Bilinear Model-based classifier stems from the fact that motions performed from different subjects share some similarities between each other. The goal is to create a bilinear model from a pool of subjects, so that it can be used to extract motion-dependent factors (for the mathematical derivations to create a bilinear model, see [9]). Then, these motion-dependent factors are used to predict data from new subjects. The general procedure for training a Bilinear Model-based classifier is as follows. For a group of subjects S1 to S*k*, a single bilinear model is created. Then, motion-dependent factors are extracted from this bilinear model, which are then used as new features to train a PM using, for example, an artificial neural network. Then, the data of a new Subject S*n* (the new user) are divided into two subsets of data, one containing the information of one single motion, and the rest containing data of all of the remaining motions. After that, using the motion-dependent factors extracted from Subjects S1 to S*k*, and the subset of data of one single motion from Subject S*n*, a new bilinear model is created for Subject S*n*. This bilinear model is then used to extract motion-dependent factors using the remaining subset of data from Subject S*n*, which contains information of all of the remaining motions. Finally, these newly motion-dependent factors are used as features to test the PM obtained at the beginning of this process.

**Figure 6:**
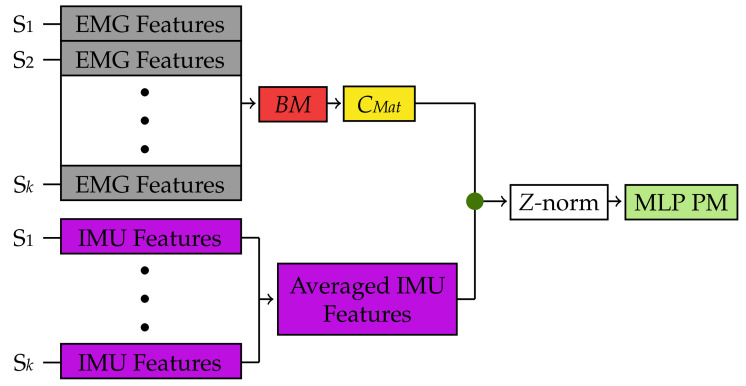
Training and testing flow for the Bilinear Model-based classification method during each cross-validation iteration using both EMG and IMU data. A similar procedure was used for the EMG only dataset. EMG data (grey) from each Subject S1 to Subject S*k* in the training folds are stacked together to form a single matrix. A bilinear EMG model (red) is created from this matrix and then, motion-dependent factors (yellow) are extracted from the bilinear model. These motion-dependent factors are fused with the averaged IMU features from the same subjects (purple) in the training folds. Finally, an MLP predictive model (green) is created using the fused data. The green dots represent a feature-level fusion.

**Figure 7:**
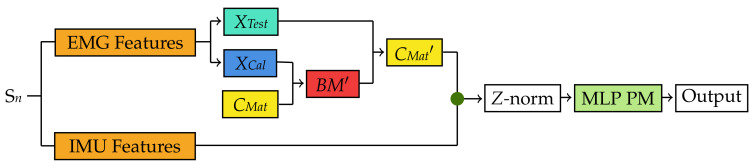
Testing flow for the Bilinear Model-based classification method during each cross-validation iteration using both EMG and IMU data. A similar procedure was used for the EMG only dataset. EMG data from Subject S*n* in the cross-validation testing fold (orange) are split into *XCal* and *XTest*. Then, a new bilinear EMG model *BM*${}^{\prime }$ (red) specific to Subject S*n* is created based on *XCal* and the motion-dependent factors extracted in Figure 6 [9]. This new bilinear model is used to estimate a new set of motion-dependent factors *CMat*${}^{\prime }$ based on *XTest* (aqua) from Subject S*n*. These motion-dependent factors are then classified using the MLP predictive model created in Figure 6. The green dots represent a feature-level fusion.

With this in mind, the Bilinear Model-based classifier was implemented using the procedure described in Figure 6 and Figure 7.

First, the EMG signals from all of the 22 subjects were re-segmented by manually selecting the indices of the onset and offset of the motion. Then, following a similar procedure as in Section 2.4, the onset and offset of the motion for the IMU’s accelerometer and gyroscope were determined. After that, time domain features were calculated from the active regions of the EMG and IMU data using the same procedure described in Section 2.5.

After the re-segmentation step, and during each cross-validation iteration, the EMG feature matrices from each subject in the training folds were vertically concatenated to form a single feature matrix (Figure 6) as indicated in [9]. Then, an EMG bilinear model *BM* was formed using Equation (4) and the method proposed by Matsubara et al. [9]. For this study, the parameters *I* and *J*, which determine the dimensions of the style and content factors, were selected as 2 and 3, respectively, as suggested in [9]. After creating the EMG bilinear model, the motion-dependent factors were extracted in the form of a content matrix, *CMat*. Furthermore, in order to explore the effects of the IMU for classifying gestures using the Bilinear Model-based classifier, *CMat* was fused with the IMU features obtained after re-segmentation. However, because these features belonged to multiple users, the average of these features across all subjects in the training folds was employed (e.g., the WL feature computed for the acceleration data in the *x* direction was averaged across all subjects) as shown in Figure 6.

Given that the Bilinear Model-based classification method requires a standard classification algorithm, an MLP network PM was created using the TensorFlow [27] library for Python [28]. The MLP network architecture consisted of the input layer, two hidden layers, and the output layer. Moreover, the two hidden layers consisted of 50 and 20 nodes, respectively. After each hidden layer, a dropout regularization layer, with a dropout rate of 20%, was included to prevent overfitting [29]. Furthermore, a batch normalization layer was added after the first dropout layer to reduce the covariate shift, i.e., the change in the distribution of the layer’s input data during training [30]. To compute the outputs of each hidden layer, a rectified linear unit (ReLU) activation function was used. Similarly, a “softmax” activation function was used to compute the output of the output layer. Before training the MLP network, each row of *CMat* was standardized using the *Z* normalization (Figure 6). Then, the MLP network was finally trained over 300 iterations with an Adam optimizer [31]. This optimizer was configured to have a learning rate of 0.001, and a decay value of $1\times 10^{-6}$ to speed up the learning process. The network architecture and the hyperparameters were determined experimentally.

In order to test the MLP PM based on *CMat*, a new bilinear model *BM*${}^{\prime }$ was formed for each subject in the cross-validation iteration testing fold. To do so, EMG data from Subject S*n* in the testing fold were divided into two subsets *XCal* and *XTest* (Figure 7). However, instead of using two repetitions of each gesture to form *XCal* as it was done in Section 3.1, only one random repetition of the wrist flexion gesture in a random arm position was used. This was based on the study by Matsubara et al. where they showed that only one motion can be used to estimate the subject-dependent factors (*z* in Equation (4)) of a new user. Using *XCal* and the *CMat* derived in previous steps, the bilinear model *BM*${}^{\prime }$ was created for Subject S*n* in the testing fold using the steps outlined in [9] (Figure 7). Furthermore, the computed parameters from *BM*${}^{\prime }$ were then used with *XTest* to derive a new content matrix *CMat*${}^{\prime }$, which was then *Z* normalized and used for testing the MLP PM created in the previous step (Figure 6). Finally, to observe the effects of the sensor fusion technique, the IMU features were fused with *CMat*${}^{\prime }$ before testing the performance of the MLP PM.

### 3.3. Classic MLP Network

**Figure 8:**
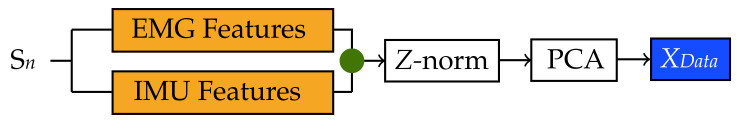
Testing flow for the classification methods during each cross-validation iteration using both EMG and IMU data. A similar procedure was used for the EMG only dataset. Data from Subject S*n* in the cross-validation testing fold (orange) are normalized and reduced to form a new dataset *XData*. The green dots represent a feature-level fusion.

**Figure 9:**
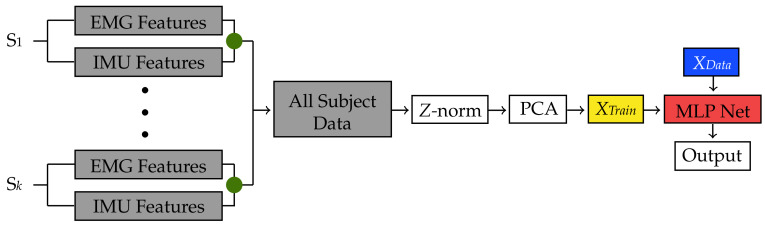
Training flow for the classification methods during each cross-validation iteration using both EMG and IMU data. A similar procedure was used for the EMG only dataset. Fused EMG and IMU data from Subject S1 to Subject S*k* in the iteration training folds (gray) are stacked together to form a unique dataset, which is then normalized and reduced to form *XTrain* (yellow). *XTrain* is then used to train a MLP network (red), which then classifies *XData* from Subject S*n*. The green dots represent a feature-level fusion.

The final user-independent classification method implemented was based on a classic MLP network classification used in our previous study [15] (Figure 8 and Figure 9). However, differently to the previous classification methods, another approach was followed for its implementation. During each cross-validation iteration, each subject’s EMG data in the training folds were combined into a single dataset by vertically concatenating their feature matrices (Figure 9). This procedure was repeated for the combined EMG and IMU dataset for further comparison. Then, this dataset was standardized and reduced using the same procedure explained in Section 3.1. It is important to mention that the data labels were also standardized as part of the process for training the MLP network. The mean and standard deviation parameters were saved to reconstruct the original labels after training the network.

To train the MLP network, a stochastic gradient descent (SGD) learning algorithm was employed during the backpropagation step. Further, the MLP network was trained in RStudio [32] with the RSNNS package software [33] as this was the software used in the preliminary study [15] described in Section 1. The procedure followed to build this MLP network is described below.

First, the MLP network dataset was split into two sub-datasets, the training and cross-validation set, each one formed by 80% and 20% of the original data, respectively. To determine the architecture of the MLP network, as well as the learning parameters, the cross-validation set was used to observe the efficiency of the model for classifying seven gestures. Using this cross-validation set, the optimal architecture of the network was found. This architecture consisted of the input layer, three hidden layers, and the output layer. The three hidden layers contained 300, 200, and 100 nodes, respectively.

Furthermore, the learning rate of the SGD algorithm was found to be 0.2. In addition, a logistic activation function was employed for the input and the three hidden layers, whereas for the output layer, a linear function was utilized as the labels were not bounded after standardization, i.e., they were not integer numbers. Finally, the output of the network was unscaled using the previously obtained mean and standard deviation parameters from the training set. However, because the values of the unscaled labels were not integer numbers, a function was employed to round them to the nearest integer so that they lay within the range of 1 to 7. After training the MLP network PM, its performance was tested using the data from each subject in the cross-validation iteration testing fold as shown in Figure 9.

### 3.4. Statistical Analysis

Following the analysis of the performance of each individual classification method, a statistical analysis was performed using the Statistical Package for Social Sciences v.25 (SPSS) software in order to identify the best classification method. First, a 3×2 (three classification methods and two sensor modalities datasets) repeated measures univariate ANOVA followed by a post hoc test with Bonferroni correction was performed to identify differences between each classification method. This was done to observe the effects that the classification method and the sensor modality (i.e., EMG data only, and EMG and IMU data) had on the gesture recognition accuracy. Furthermore, a simple main effect analysis was performed in order to observe the interaction between the recognition accuracy at each level of the classification method and the sensor modality. It is important to mention that the test for normality was performed using the Shapiro–Wilk test, and given that the data were found not to be normal, the non-parametric Friedman test was used to analyze the data. However, given that the results of the Friedman test were similar to those of the three way repeated measures univariate ANOVA mentioned before, it was decided to use the parametric test for the statistical analysis. Furthermore, all of the statistical analyses were performed using a Greenhouse–Geisser correction, as the results from the Mauchly’s test of sphericity were significant (p=0.046).

## 4. Results

The accuracy outcomes of each user-independent classification method were obtained after classifying data from the seven gestures using two different sensor modalities: EMG, and EMG and IMU. Classification results from each cross-validation iteration defined in Section 2.6 using each classification method are presented in Table 2.

**Figure 10:**
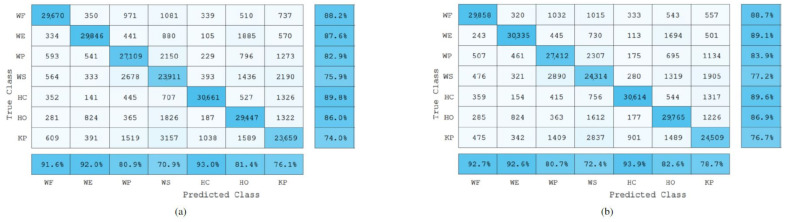
Adaptive LS-SVM confusion matrices of the seven gestures collected using EMG data only (**a**), and EMG and IMU data (**b**) from all testing subjects. The numbers inside each cell represent the number of sample points classified. The last row from each confusion matrix represents the precision score percentages of each class. Similarly, the last column represents the recall score percentages of each class.

**Figure 11:**
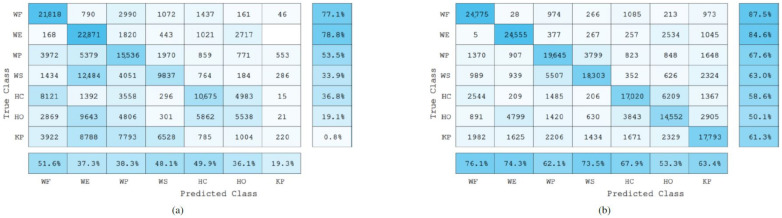
Bilinear models-based confusion matrices of the seven gestures collected using EMG data only (**a**), and EMG and IMU data (**b**) from all testing subjects. The numbers inside each cell represent the number of sample points classified. The last row from each confusion matrix represents the precision score percentages of each class. Similarly, the last column represents the recall score percentages of each class.

**Figure 12:**
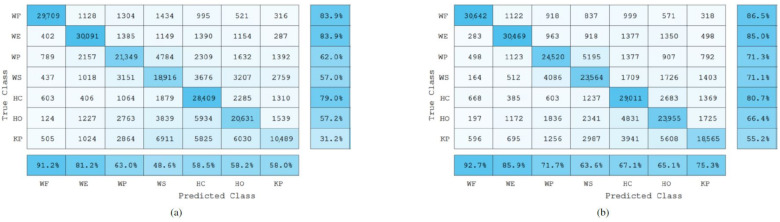
MLP networks confusion matrices of the seven gestures collected using EMG data only (**a**), and EMG and IMU data (**b**) from all testing subjects. The numbers inside each cell represent the number of sample points classified. The last row from each confusion matrix represents the precision score percentages of each class. Similarly, the last column represents the recall score percentages of each class.

Furthermore, the precision and recall scores were also calculated for each gesture. Precision evaluates the performance of the model on the positive class, i.e., it highlights the ability of the classification model to return only relevant data, or it shows which classified gestures actually belong to a specific class. On the other hand, the recall value represents the rate of the true positives (the correctly classified samples) of a specific class when compared against the false negatives, which are the samples that were incorrectly classified as a different class. Figure 10, Figure 11 and Figure 12 show the confusion matrices of the total data of each gesture (approximately 32,560 sample points per gesture) from the 22 testing subjects when classifying the gestures in a user-independent scenario using EMG data only, and a combination of EMG and IMU data.

### 4.1. Adaptive LS-SVM Classification Results

A significant difference (p<0.01) was observed between the mean recognition accuracy of the combined EMG and IMU data (84.6%) and the mean recognition accuracy of the EMG data only (83.5%) when using the Adaptive LS-SVM classification method. This significant difference can be further explained by the high correlation that exists between the two sets of classification accuracies for each of the 22 subjects, with an almost perfect $R^{2}$ of 0.9856. This high correlation translates into a low variability within each subject, which results in a reduced mean squared error term when computing the *F*-statistic in the ANOVA analysis. However, even though a significant statistical difference exists, the effect of the combined EMG and IMU sensor modality was low, as shown by the eta-squared ($\eta^{2}$) obtained by the simple main effects analysis of the sensor modality, which was equal to 0.607.

Finally, from the confusion matrices in Figure 10, it can be observed that both, the precision and recall scores, had an overall increase when classifying data from the combined EMG and IMU data. This highlights the ability of the classification method to recognize each individual gesture better.

### 4.2. Bilinear Models-Based Classification Results

Similarly to the Adaptive LS-SVM classification model, the results obtained by using EMG based bilinear models in combination with the IMU sensor data showed a significant increase (p<0.01) in the overall gesture recognition accuracy (67.5%) when compared to the recognition accuracy obtained by using the EMG bilinear model only (42.8%).

Furthermore, from the confusion matrices in Figure 11, it can be observed that when using the data coming from the IMU, the classification model was able to increase the recall scores. For example, from the confusion matrix in Figure 11a, it can be seen that the Key Pinch gesture was misclassified most of the time as being a Wrist Extension or a Wrist Supination gesture. Therefore, by adding the IMU data, the NN algorithm employed was able to cope with this issue by using the information from the extra features to make more general assumptions about the corresponding class of unseen data.

### 4.3. Classic MLP Network Classification Results

Finally, the classification performance of the MLP networks improved when using a combination of the EMG and IMU sensor data. In this sense, the mean recognition accuracy when using the two-sensor modality (73.7%) was significantly different (p<0.01) from the mean recognition accuracy obtained when using the EMG data only (64.8%). Furthermore, an increase in the precision and recall scores can be observed from Figure 9. This indicates that the MLP network was able to overcome the intrinsic variability of the EMG signals among different subjects.

### 4.4. Comparison of Classification Methods

Table 3 shows the statistical analysis results of comparing the three classification models using the EMG and IMU sensor modality. A statistical significance was found between all three models (p≤0.001), which means that the classification performance is not only affected by the sensor modality as shown above, but also by the type of classification approach employed. Furthermore, it can be noted that the best classification method was the Adaptive LS-SVM. Interestingly, the classification method that performed second best was the one based on the classic MLP network, which suggests that methods that do not require transfer of prior knowledge, such as the MLP network, are able to generalize better to unseen data. This shows an advantage over the Adaptive LS-SVM and the Bilinear Model-based classification algorithms that require information from at least one motion (*XCal* in Section 3.1 and Section 3.2) to accurately classify data.

**Figure 13:**
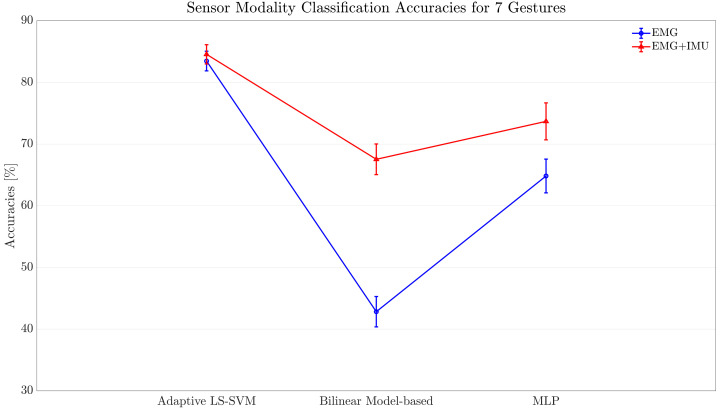
Overall accuracies of the classification methods using EMG data, and EMG and IMU data. Error bars represent the standard error of the mean.

Figure 13 shows the classification performance when classifying both the EMG, and EMG and IMU data using each classification method. A significant interaction was observed between all of the three classification methods and the two sensor modalities. In this sense, it can be seen that out of the three classification methods using EMG data only, the one that performed best was the Adaptive LS-SVM. However, when classifying a combination of EMG and IMU data, the Bilinear Model-based classification algorithm and the MLP networks were able to significantly increase their performance, being the Bilinear Model-based classification algorithm the one that benefited the most of the sensor fusion technique when compared against the other two methods.

## 5. Discussion

### 5.1. Adaptive LS-SVM Classification

The results obtained from using a sensor fusion approach in the Adaptive LS-SVM classification method indicate that by combining the EMG and IMU features, not only did the classification performance improve, but also the ability of the model to recognize each individual gesture. Furthermore, the addition of the IMU features did not affect the behavior of the Adaptive LS-SVM classification method. In this sense, the distribution of the data among subjects was not affected, which is why the difference between the mean classified accuracies of the EMG, and the EMG and IMU datasets was of 0.956%. However, similar to what Tommasi et al. [8] found, there were subjects whose recognition accuracy performed worse than others. This was because their data distribution was not able to match those of the pretrained models, thus preventing transfer of prior knowledge. This indicates that information from more subjects needs to be recorded in an attempt to compensate for this issue.

These results suggest that the Adaptive LS-SVM classification based on the combination of EMG and IMU sensor data can be effectively used in a user-independent scenario. However, as explained before, data from more subjects need to be recorded to have a larger database of pretrained models. By doing so, it will be easier to match the data distribution of new users to one of these pretrained models.

### 5.2. Bilinear Models-Based Classification

Although the classification performance improved after combining the EMG and IMU data, the low recognition accuracies impose a major drawback on the EMG Bilinear Model-based classification method. This poor performance can be explained by three important factors with the first one being the classification under confounding factors, i.e., the gestures being classified under different arm positions. To deal with this issue, Ishii et al. [34] proposed the use of a two-stage bilinear model in which during the first stage, user-dependent and postural-dependent factors were separated. Then, during the second stage, the motion-dependent factors were separated from the postural-dependent factors. However, even though the results showed an improvement during classification, the validation of their methods did not provide enough details. In this sense, they failed to report the procedure followed to build the bilinear models, e.g., the number of subjects assigned to the training and testing set or the classification algorithm parameters used were not reported. Further, no statistical analysis was performed to validate their approach.

The second important factor that affected the classification performance of the proposed method, was the donning of the Myo Armband by novel users. As explained by Matsubara et al. [9], the use of bilinear models required the electrodes to be placed on the exact same location for all of the users. However, the difference in the dimensions of the forearm between subjects imposes a great obstacle for achieving this requirement. This suggests that the classification method can be further optimized by adopting a similar approach as in [35], were the displacement of the sensors was estimated in order to improve the classification accuracies.

Finally, the third factor was related to how the style and content variables were computed. The values used in this study for the parameters *I* and *J* resulted in a reduced number of features used for classification, which could have increased the bias of the MLP network algorithm. To cope with this issue, a similar approach used to obtain the principal components during PCA could be applied. Given that the iterative process to find the style and content variables is based on using the singular value decomposition (SVD) factorization [9], the values *I* and *J* can be initialized as the *n*th row of the diagonal matrix sigma computed for each variable, with *n* being small enough, so that a specific percentage of the variance is retained.

Overall, the inclusion of the IMU features as an extra input for the classification of gestures using bilinear models showed that this classification method has the potential to be used in a user-independent scenario. However, further improvement needs to be done to increase the efficacy of the proposed method in order to improve the recognition accuracies.

### 5.3. Classic MLP Network Classification

Despite being able to increase the recognition accuracy and keep a consistent classification accuracy trend with new subjects, the classic MLP Network classification approach has several limitations. It is well known that the topology of an ANN is usually determined empirically, so by adopting an approach similar to that of Lima et al. [26], where a hybrid intelligent system that combined ANN with optimization algorithms was developed, the performance of the proposed model could be improved. Another limitation is the improper placement of the Myo Armband, which can affect the performance of the classification algorithm, specially in a user-independent scenario. To deal with this issue, the approach followed by Allard et al. in [36] can be implemented, in which a classifier was trained using the data obtained from the Myo Armband while it was placed in different forearm locations. By doing so, it may be possible to avoid the change of distribution of the data due to displacements of the EMG electrodes. One final limitation of the proposed classification method is that MLP networks are sensitive to feature noise, which is attributed to the inability of the users to match the same level of contraction when performing the trained gestures [37]. This will inevitably degrade the ability of the trained network to properly classify the gestures, thus requiring the MLP network to be retrained at some point. Therefore, a self-recalibration algorithm that can track said degradation (e.g., [38]) can be implemented to improve the robustness of the classifier.

Overall, the results showed that it is possible to achieve recognition accuracies of up to 88.3% with an average recognition accuracy of 73.7% among five subjects when classifying seven gestures using a combination of EMG and IMU features. This indicates that by using the proposed classification approach, which consists of classifying EMG and IMU data coming from the Myo Armband using MLP networks, it is possible to achieve a user-independent classification.

## 6. Conclusions and Future Work

The work presented in this study was aimed towards developing a user-independent hand gesture recognition classification model using IMU and EMG-based sensor fusion techniques. The purpose of the study was to improve existing user-independent classification models that relied solely on classifying EMG data. To achieve this goal, each existing user-independent classification model was compared against each other to find the best model before and after applying the EMG and IMU sensor fusion techniques.

User-independent classification methods were created in an attempt to accelerate the pattern recognition training times for end-users. By doing so, the user’s learning process of the control of wearable mechatronic devices would be reduced, thus promoting long term adoption of this technology. However, even though these methods have shown promising results, they are still at an early stage [10]. This study attempted to enhance some popular user-independent classification methods, which included some adaptive learning frameworks, by employing data gathered from all of the sensors embedded in the Myo Armband. The standard methods followed by other pattern recognition algorithms were applied. In this sense, EMG and IMU data from healthy subjects, who performed seven hand and finger gestures in four arm positions, were collected and processed, and a set of features was extracted from these data. Then, existing EMG-based user-independent classification models were improved by adding information from the IMU. As a result, accuracies of up to 92.9% were achieved for the best performing model (Adaptive LS-SVM). Additionally, a statistical analysis was performed to compare the effects of adding the IMU data to each of the user-independent classification models. In general, all of the tested models improved their classification accuracy significantly.

Although this work showed that the classification performance improved the user-independent classification methods after adding the information collected from another sensor, there is room for further improvement. Future work will focus on the improvement of these classification methods using different types of sensor fusion levels such as data-fusion level and decision-fusion level. Furthermore, the addition of more user-independent classification algorithms will be explored for further comparison. One interesting classification algorithm is the one presented by Khushaba [39], in which a canonical correlation analysis was presented to adapt pretrained EMG models to new users. Interestingly, this method could prove to be useful, as the canonical correlation analysis is invariant to electrode position, an issue presented by the Bilinear Model-based classification algorithm. In addition, the user-independent classification algorithms presented in this study using sensor fusion techniques will be used in a stroke population and in an online setting. To this end, it will be important to improve the motion onset detection algorithm presented in this study. Furthermore, it is worth mentioning that the classification of different hand and finger movements not mentioned in this study could prove useful for certain applications. For example, when picking small objects, it would be best to use a precision pinch [15] instead of a hand-closed gesture. Future work should focus on the effect of new gestures on the performance of the user-independent classification methods. Another potential venue to explore could be the use of different motion onset and offset detection methods, as the one described in Section 2.4 that relies heavily on the amount of force exerted during gesture performance. For example, it would be worth exploring the use of the approximated generalized likelihood ratio, as it is known to have good detection for smooth motions such as the ones presented in this study [40]. Finally, all of these recommendations should be implemented using a different data collection hardware that could be robust and commercially available, as it is known that development for the Myo armband is currently suspended.

## Figures and Tables

**Table 1:** Summary of participant demographics.

Sex	Dominant Hand	Age (yrs)	Weight (kg)	Height (cm)	Wrist Circumference (cm)	Forearm Circumference (cm)
18 Male	22 Right	23.70 ± 3.92	71.30 ± 12.13	173.67 ± 10.51	16.42 ± 1.20	26.41 ± 2.81
6 Female	2 Left					

**Table 2:** Pairwise comparison of the EMG, and EMG and IMU datasets for each classification method. The *p* value represents the significance level of the pairwise comparison between the mean recognition accuracy of the sensor modalities on a specific classification model. Numbers in brackets represent the standard deviation.

	EMG		EMG + IMU		
Classification Method	Accuracy Range (%)	Mean Accuracy (%)	Accuracy Range (%)	Mean Accuracy (%)	*p* Value
Adaptive LS-SVM	61.7–92.5	83.5 (±7.5)	62.5–92.9	84.6 (±7.3)	<0.01
Bilinear Model-based	21.2–67.3	42.8 (±11.5)	43–84.9	67.5 (±11.6)	<0.01
MLP Network	36.5–78.9	64.8 (±12.8)	36.1–88.3	73.7 (±14.1)	<0.01

**Table 3:** Pairwise comparison of the different classification methods using a combination of EMG and IMU data.

Classification Method		Mean Difference (%)	Std. Error (%)	Significance
Adaptive LS-SVM	Bilinear Models	28.827	2.508	<0.001
	MLP Networks	14.751	3.230	0.001
Bilinear Models	MLP Networks	−14.076	2.077	<0.001

## Data Availability

The data obtained in this study have not been approved by the Research Ethics Board for open access and are therefore not available to the public.
